# Continuous Positive Airway Pressure Improves Marital Relationship and Sexual Satisfaction in Obstructive Sleep Apnea Patients and Partners

**DOI:** 10.1111/jsr.70167

**Published:** 2025-08-21

**Authors:** J. Chorvoz, C. Rabec, M. Labruyère, H. Devilliers, P. Sabot, A. Berrier, D. Schenesse, P. Mouillot, P. Bonniaud, M. Georges

**Affiliations:** ^1^ Department of Pulmonary Medicine and Intensive Care Unit, Constitutive Reference Center for Rare Pulmonary Diseases Dijon‐Bourgogne University Hospital Dijon France; ^2^ Faculty of Medicine and Pharmacy University of Burgundy Dijon France; ^3^ Department of Intensive Care Dijon‐Bourgogne University Hospital Dijon France; ^4^ Department of Internal Medicine and Systemic Diseases Unit Dijon‐Bourgogne University Hospital Dijon France; ^5^ Clinical Investigation Center INSERM CIC‐EC 1432, Dijon‐Bourgogne University Hospital Dijon France; ^6^ INSERM LNC UMR 1231, LipSTIC LabEx Team Dijon France; ^7^ Centre des Sciences du Goût et de l’Alimentation UMR 6265 CNRS 1234 INRA, University of Burgundy Dijon France

**Keywords:** continuous positive airway pressure, dyadic relationship, obstructive sleep apnea, quality of life, quality of sleep, sexual satisfaction

## Abstract

Symptoms of obstructive sleep apnea and hypopnea can interfere with sleep and quality of life for the patient's partner and affect marital relationships. Our primary objective was to assess how continuous positive airway pressure (CPAP) treatment affects couples using the Dyadic Adjustment Questionnaire (DAS‐16). Secondary objectives focused on the effects on the dyad's quality of life and sleep, depending on the technical characteristics of the device. Prospective monocentric study including consecutive couples in which one of the partners began treatment by CPAP between July 2022 and February 2024 at Dijon University Hospital. After inclusion, dyads were evaluated 2 and 6 months after treatment initiation. Fifty‐four couples were analysed. After 2 months under CPAP, the dyadic adjustment score (DAS‐16) significantly improved in partners (ΔDAS‐16 = +1 [−1; 3], *p* = 0.025) and in patients (ΔDAS‐16 = +2 [−1; 5], *p* = 0.002). DAS‐16 improvement was stable after 6 months. Sexual satisfaction followed the same trend. Sleep quality assessed by the Pittsburgh Sleep Quality Index (patients) and the Athens Insomnia Scale (partners) improved significantly, as did daytime sleepiness. A significant reduction in anxiety and depression scores, as well as an improvement in several domains of the SF‐36 questionnaire, were also demonstrated in both groups. Our study shows that the use of CPAP improves the quality of marital relationships, as perceived by both partners. These findings could provide clinicians with additional tools to enhance patient adherence and compliance with therapy.

**Abbreviations**
AHI: apnea‐hypopnea index
AIS: Athens Insomnia Scale
BMI: body mass index
CPAP: continuous positive airway pressure
DAS‐16: Dyadic adjustment scale in 16 questions
ESS: Epworth sleepiness scale
FEV1: forced expiratory volume in one second
HADS: hospital anxiety and depression scale
ISS: index of sexual satisfaction
LSEQ: Leeds sleep evaluation questionnaire
ODI: oxygen desaturation index
OSA: obstructive sleep apnea
PPI: positive psychological intervention
PSQI: Pittsburgh sleep quality index
SF36: short form 36 health survey
SpO2: haemoglobin oxygen saturation by pulse oximetry
TIA: transient ischemic attack
VAS: visual analogic scales
VC: vital capacity

## 1 Introduction

Obstructive sleep apnea (OSA) is a highly prevalent disease affecting up to 49% of men and 23% of women; depending on the definition of OSA used and the characteristics of the population studied, almost 425 million adults worldwide require treatment (Benjafield et al. 2019). These rates have substantially increased over the last two decades due to the associated risk factors (e.g., obesity and ageing). OSA is characterised by recurrent episodes of partial or complete obstruction of the upper airways during sleep, leading to poor sleep quality (Destors et al. 2017; Lévy et al. 2015). Sleep fragmentation has a variety of medical and functional consequences including excessive daytime sleepiness, mood disturbance, impaired cognitive performance and altered quality of life (Gottlieb and Punjabi 2020; Lévy et al. 2015). Among the nocturnal symptoms of OSA are snoring, disrupted sleep, changes in body position or agitation. Because sleep is a shared experience for 61% of adults (Rosa et al. 2022; Richter et al. 2016), partners are also likely to experience sleep disturbances (Rosa et al. 2022; Luyster 2017), which may contribute to daytime impairments of physical and mental health and relationship problems involving couple functioning, marital satisfaction and social interactions (Acar et al. 2016). Snoring, a hallmark symptom of OSA, can be a major burden for partners (Lugaresi et al. 1982), requiring the use of earplugs or even separate sleeping quarters (Richter et al. 2016) and causing anxiety due to perceived breathing arrests during the night (Rosa et al. 2022). Sexual dysfunction (Liu et al. 2015), with a loss of interest in intimate relations and an increase in erectile dysfunction in men (Andrianne and Legros 2010; Gu et al. 2022; Lai et al. 2016) are other typical symptoms that decrease quality of life for patients and their partners (Rosa et al. 2022).

Continuous positive airway pressure (CPAP) therapy is the first‐line treatment for OSA (Pavwoski and Shelgikar 2017), and ≥ 4 h daily use is a frequently cited threshold for achieving optimal therapeutic benefit (Portier et al. 2010; Gagnadoux et al. 2011; Kribbs et al. 1993; Medicare Coverage Database 2021). However, adequate CPAP adherence is problematic (Rotenberg et al. 2016; Pépin et al. 2021), and up to 50% of patients with OSA refuse CPAP or discontinue it within the first week (Rosa et al. 2022). Up to 60% of patients who initially accept CPAP treatment are not fully adherent despite the use of increasingly comfortable, silent, and compact equipment and the various therapeutic education interventions (Rotenberg et al. 2016; Young et al. 2002). Prior to CPAP initiation, OSA patients can be worried that a CPAP device would make them less attractive to their partner. However, CPAP may improve partner sleep quality and daytime functioning (e.g., mood, daytime sleepiness, cognitive function, quality of life, work performance, relationship quality) (Luyster 2017; Mehrtash et al. 2019). Moreover, several studies have highlighted the influence that partners can have on health behaviours. The support of a partner is a key factor for adherence to chronic disease treatment (Luyster 2017; Gagnadoux et al. 2011). Little is known about the role of couple relationships in CPAP adherence, and available studies have discordant results suggesting that collaborative partner involvement has positive or negative effects on CPAP use (Luyster 2017; Baron et al. 2009, 2011; Gentina et al. 2019). Examining the dyadic perspective is critical to gaining a better understanding of factors that improve adherence and treatment outcomes (Luyster 2017; Wozniak et al. 2014; Antoine et al. 2008; Wawrziczny et al. 2022).

The main goal of our study was to assess the impact of CPAP therapy on the relationship of OSA patients and partners of both genders. We also assessed the impact of CPAP therapy on sexual satisfaction, partners' sleep and daytime functioning.

## 2 Methods

### 2.1 Patients and Study Design

Patients with a confirmed diagnosis of moderate to severe OSA by nocturnal ventilatory polygraphy, requiring CPAP therapy, according to current French guidelines (Portier et al. 2010), and their partners from were included in this prospective, single‐center, open‐label study from May 2022 to February 2024 at the Sleep Center of the Pneumology and Respiratory Intensive Care Department of Dijon University Hospital. The inclusion criteria stipulated that both partners were over 18 years of age, had been living together for over a year, could speak and read French, had provided oral informed consent, and were affiliated to the national health insurance system. We excluded couples if patients or their partners were older than 80 years, pregnant, had previous treatment with CPAP or prior diagnosis of other sleep disorders, did not share their bed for reasons other than OSA discomfort, were unable to give informed consent or to complete the questionnaires due to previous psychiatric or neurological disorders such as dementia, psychosis, or severe mental disability.

### 2.2 CPAP Administration

All patients who had a scheduled appointment for CPAP therapy initiation were contacted by phone by the principal investigator (JC) who offered the opportunity to participate in this study. After they gave their verbal consent, patients were asked to come to a baseline visit (M0) with their partner. CPAP therapy was initiated by an experienced nurse. All patients received treatment education including explanation of the treatment to encourage adherence, mask‐fitting and a CPAP acclimatization period during the daytime. CPAP treatment was provided with an automated positive airway pressure machine (CPAP S1, Resmed, San Diego, US) that was initially set in automatic mode (from 4 or 6 cmH_2_O to 14 cmH_2_O) with a nasal mask if well tolerated. Patients and partners completed questionnaires. The efficacy and compliance of nocturnal CPAP treatment were verified by analysis of home CPAP oximetry and corresponding detailed ventilator data after one week. The use of long term fixed or auto adjusted CPAP treatment was decided by the treating physician based on device data and patient tolerance. If a fixed pressure was chosen, the 95th percentile of pressure was used as reference. Heated humidification was added when nasal side effects of CPAP were reported. A chin strap or an oro‐nasal mask were offered to patients with major mouth leaks under CPAP. After 2 months of treatment, all patients had a follow‐up visit and the efficacy of nocturnal CPAP treatment and compliance were again verified. Patients and partners completed questionnaires. After 6 months (M6), the participants were contacted by phone. Compliance and efficacy were checked using CPAP telemonitoring. Questionnaires were sent to be completed at home with a pre‐stamped envelope.

### 2.3 Data Collection

Prior to CPAP initiation, details of current marital behaviours were documented through a structured questionnaire including marital status, number of children at home and sleeping habits. The primary outcome was based on the results of the Dyadic Adjustment Scale (DAS‐16), a 16‐item self‐questionnaire, scored from 1 to 6 on a Likert scale. A dyad refers to two people who have influence each other. This questionnaire is used to assess the degree of agreement and the quality of the dyadic interactions in the couple. The DAS‐16 scale ranges from 16 (the poorest score) to 96 (the best score). Partners also completed the Index of Sexual Satisfaction Index (ISS), a 25‐item self‐ questionnaire (Wawrziczny et al. 2022; Comeau and Boisvert 1985). The ISS scale ranges from 0 to 100, with the lowest scores indicating sexual dissatisfaction within the couple.

Baseline and each follow‐up visit also included recording of subjective sleep quality over the last month using the Pittsburgh Sleep Quality Index (PSQI) (Blais et al. 1997), the Leeds Sleep Evaluation Questionnaire (LSEQ) (Tarrasch et al. 2003) and the Athens Insomnia Scale (AIS) (Soldatos et al. 2000). Additionally, all participants filled in the Epworth Sleepiness Scale (ESS) (Johns 1991) to evaluate daytime drowsiness and the Short Form 36 Health Survey questionnaire (SF‐36) evaluating health‐related quality of life (Ware and Gandek 1998). Finally, the Hospital Anxiety and Depression Scale (HADS) was used to screen partners for underlying anxiety and depressive disorders (Zigmond and Snaith 1983).

Self‐administered visual analogic scales (VAS) were developed by a consensus of experts (C.R., M.G., P.S., J.C.) to evaluate the discomfort and stress generated by the apnea episodes during the partner's sleep, satisfaction with marital life, and sexual desire for the partner before and during CPAP treatment. It also assesses the partner's tolerance of CPAP device (noise, leaks) and the patient's autonomy in fitting the mask. The VAS is a 100‐mm line joining two opposing statements. Before completing the VAS, the participants were instructed to draw a cross on the line to represent their feelings over the past week.

At M2 and M6, data recorded by the built‐in CPAP software were collected (AHI, compliance and leaks as stipulated by manufacturer). OSA symptoms, tolerance and adherence to CPAP therapy, and mask comfort were assessed. CPAP effectiveness was defined as follows: (1) an index of residual obstructive events under CPAP (residual AHI) reported by the integrated built‐in software less than five events per hour of recording, (2) normalisation of nocturnal pulse oximetry (mean SpO2 ≥ 90%, oxygen desaturation index < 5 events/h), (3) mean daily CPAP use of at least 4 h per night on 70% of nights during the last consecutive 30 days (Medicare Coverage Database 2021) and (4) lack of major side effects.

### 2.4 Statistical Analysis

A preliminary study observed a significant increase of 3 ± 5 points in the DAS‐16 score (Antoine et al. 2008). We estimated that with a sample of 34 patients, the study would have 80% power to demonstrate an improvement in the DAS‐16 of 3 ± 6 points after 2 months of treatment with CPAP using a one‐sided type I error rate of 5%. We planned to include 50 couples to anticipate patients lost to follow‐up, with suboptimal CPAP effectiveness. Analyses were performed using XLSTAT software version 25.2.0 (14.11) and R version 4.4.2.

All the variables studied are described as medians and interquartile ranges for quantitative variables, and numbers and percentages for qualitative variables at the different measurement times.

The normality of the distribution was tested using the Shapiro–Wilk test. Many variables do not follow a normal distribution, so comparisons were made using the Wilcoxon signed rank test. The assumption of normality was satisfied, so a repeated measures analysis of variance (ANOVA) was conducted to assess changes in quality of life and sleep scores over time within the same group of participants. Prior to the analysis, the assumption of sphericity was tested using Mauchly's test. Post hoc pairwise comparisons were performed using the Bonferroni correction to identify significant differences between specific time points.

Following the ANOVA, a principal component analysis (PCA) was conducted to address the issue of multicollinearity and reduce the dimensionality of the dataset, thereby facilitating a more robust interpretation of the quality of life and sleep variables. The PCA was applied to the correlation matrix to standardise the data, ensuring that each variable contributed equally to the analysis, regardless of scale. The first few principal components that cumulatively explained the majority of the total variance were retained for further analysis. Each principal component was interpreted based on the loadings of the original variables, with higher absolute loadings indicating a stronger relationship between a variable and the corresponding component. The scree plot and cumulative variance explained were also examined to confirm the number of components to retain. This dimensionality reduction allowed us to highlight the underlying structure of the data, identifying the most important factors contributing to variations in quality of life and sleep outcomes in both patients and their partners.

### 2.5 Ethical and Regulatory Aspects

This study does not constitute Research Involving the Human Person within the meaning of article R1121‐ of the French Public Health Code. Although it was organised and carried out on healthy or sick people, the purpose of the research was not to develop biological or medical knowledge with a view to evaluating the functioning mechanisms of the human organism or the efficacy and safety of procedures, but rather to carry out experiments in the human and social sciences in the field of health. As such, the study did not require authorisation from the French regulatory authorities (Gorphe and Jannin 2019).

## 3 Results

### 3.1 Study Population

**FIGURE 1:**
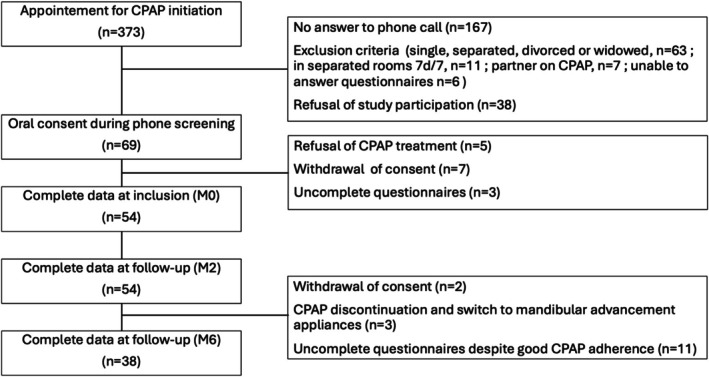
Study flow chart.

From September 2022 to December 2023, 373 patients were eligible for CPAP treatment (Figure 1). Of those, 69 dyads meeting inclusion criteria agreed to participate, and 54 answered all questionnaires at inclusion before starting nocturnal CPAP treatment. Only 38 dyads completed follow‐up.

Baseline characteristics are reported in Table 1. Patients on CPAP (78% men, median age 64 [55–69] years) were treated for severe OSA (80% of patients with AHI over 30 events per hour, median AHI of 38.5 [30–54.3] events per hour). Most patients were symptomatic. They had few cardiovascular comorbidities, except for hypertension, which affected 57% of them. At inclusion, dyads had lived together for a mean of 32 [16.5–44] years and had 2 [0–2] children who no longer lived at home. They shared the same bed 7 days a week (35% of patients had a mattress less than 160 cm wide).

**TABLE 1:** Patient characteristics at baseline (M0).

Patient characteristics at baseline	(*n* = 54)
Demographics and clinical data	
Age (years)	64 [55–69]
BMI (kg/m^2^)	29.8 [26.9–35.4]
Systolic blood pressure (mmHg)	130 [120–140]
Diastolic blood pressure (mmHg)	75 [70–81]
SpO2 (%)	96 [95–97]
Heart rate (pulses/min)	70 [63–74]
FEV1 (% of predicted value)	97 [87–111]
VC (% of predicted value)	98 [88–111]
FEV1/FVC (%)	82 [76–98]
Medical history	
Stroke	8 (15)
Heart disease	11 (20)
Atrial fibrillation	8 (15)
Obliterative arterial disease of the lower limbs	4 (7)
Hypertension	31 (57)
Dyslipidemia	17 (31)
Diabetes	10 (19)
Smoking status	
Active	7 (13)
Weaned smoker	32 (59)
Non‐smoker	17 (31)
Onset symptoms	
Snoring	45 (83)
Suffocating awakenings	20 (37)
Apneas observed by spouse	28 (52)
Restless sleep	35 (65)
Non‐restorative sleep	36 (67)
Morning headaches	20 (37)
Concentration difficulties	33 (61)
Nocturia	27 (50)
Night sweats	24 (44)
OSA severity	
Onset AHI	38.5 [30–54.3]

*Note*: Quantitative values expressed as medians [25th—75th percentile]. Qualitative values expressed as number (percentage).

Abbreviations: AHI, apnea hypopnea index; BMI, body mass index; FEV1, forced expiratory volume in 1 s; FVC, forced vital capacity; OSA, obstructive sleep apnea; SpO2, haemoglobin oxygen saturation by pulse oximetry; VC, vital capacity.

### 3.2 CPAP Treatment Characteristics

Most patients used an auto‐adjusted CPAP device on a long‐term basis and a nasal mask. Median compliance was 6 [4.5–7] hours per night; 78% of patients were compliant (Table 2). Adjustments of the CPAP device were adequate, and CPAP was effective in 72% of patients after 2 months of treatment (median residual AHI 1.6 [1–3.2] events per hour) (Table 2).

**TABLE 2:** Characteristics of CPAP treatment collected 2 months (M2) after initiation.

Treatment description	M2 (*n* = 54)
Traitement features	
Autoadjust CPAP	38 (70)
Fixed mode	16 (30)
Nasal mask	40 (74)
Oronasal mask	14 (26)
Data extracted from CPAP built‐in software	
Average compliance (hours)	6 [4.5–7]
Compliance ≥ 4 h*	42 (78)
Average residual AHI	1.6 [1–3.2]
Residual AHI ≤ 5	43 (88)
Unintentional leaks at the 95th percentile	15 [5–22]
Oximetry	
ODI < 5 events per hour	36 (67)
Average SpO2 on CPAP	94 [92.4–95]
OSA control	
Well controlled, %	39 (72)
Poorly controlled, %	15 (28)

*Note*: Quantitative values expressed as medians [25th—75th percentile]. Qualitative values expressed as number (percentage).

Abbreviations: AHI, apnea hypopnea index; CPAP, continuous positive airway pressure; M2, after 2 months of treatment; ODI, oxygen desaturation index; OSA, obstructive sleep apnea; SpO2, haemoglobin oxygen saturation by pulse oximetry.

* Per night, more than 70% of nights over the last 30‐day period.

### 3.3 Dyad Relationship

**FIGURE 2:**
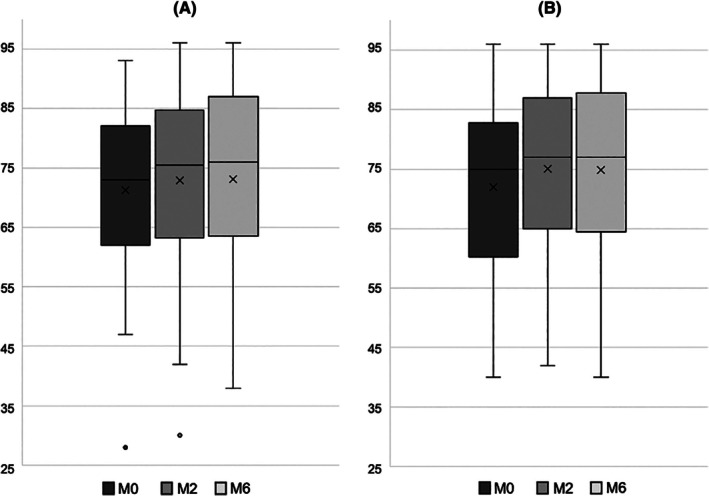
Changes in the Dyadic Adjustment Scales (DAS‐16), completed by partners (panel A) and OSA patients (panel B), between baseline (M0), two (M2) and 6 months (M6).

After 2 months of CPAP treatment, there was a significant improvement in the DAS‐16 completed by patients under CPAP (75 [60–83] at M0 versus 77 [65–87] at M2, *p* = 0.002) and their partners (73 [62–82] at M0 versus 75.5 [63–85] at M2, *p* = 0.025) (Figure 2). DAS‐16 improvement was stable after 6 months under CPAP (at M6, in patients: 77 [64.5–88]; in partners: 76 [63.5–87]).

Sexual satisfaction significantly improved in both groups during follow‐up. In OSA patients, the overall ISS score was 71 [50.3–77] at M0 versus 75.5 [58.5–90.5] at M2 (*p* < 0.001) versus 80.5 [58–94.8] at M6 (*p* < 0.001). In partners, the overall ISS score was 62.5 [48.5–78.8] at M0 versus 71.5 [57.3–87] at M2 (*p* < 0.001) versus 77 [62.3–95.5] at M6 (*p* < 0.001). However, the assessment of marital relationship using VAS may not be sufficiently sensitive to reflect this evolution during follow‐up (Table 3).

**TABLE 3:** Comparison of VAS about relationship completed by partners and OSA patients at baseline (M0), after 2 (M2) and 6 months (M6) of CPAP treatment.

	M0	M2	M6
VAS (0 to 10) completed by patients			
Are you satisfied with your marital life in general?	7.5 [6–9]	9 [7–10]*	8 [7–10]
Do you feel desire for your partner?	7 [5–9]	8 [5.3–9]	7 [5–10]
Does your partner feel more desire for you?		7 [6–9]	7 [5–8.8]
Has your CPAP changed your relationship?^a^		5 [5–5]	5 [5–6.8]
VAS (0 to 10) completed by partners			
Are you satisfied with your marital life in general?	8 [6–9]	8 [7–10]*	8 [6–10]
Do you feel desire for your partner?	7 [5–9]	8 [6–9]*	7 [5–9]
Has your partner's CPAP changed your relationship?^a^		5 [5–5]	5 [5–5]

Abbreviations: CPAP, continuous positive airway pressure; M0, baseline; M2, after 2 months of treatment; M6, after 6 months of treatment; VAS, visual analogue scale.

* *p* < 0.05, quantitative values expressed as medians [25th—75th percentile].

^a^ For this question: 0, deterioration; 5, no change; 10, improvement.

### 3.4 Sleep Quality

Self‐assessed sleep quality and sleepiness significantly improved in both groups (Table 4). Patients and partners reported a positive impact of CPAP treatment and little discomfort related to the CPAP device (Table 5). At inclusion, partners reported sleep disturbance because of partner snoring (56%), anxiety related to perceived apnea (26%), and nocturnal restlessness (17%). In 43% of cases, partners indicated that nocturnal apneas led them to wake their partner. After 2 months of CPAP use, only 4% still complained of snoring, 6% of anxiety and 6% of nocturnal restlessness. Furthermore, a reduction in earplug use (35% at inclusion versus 9% at M6) and the need for sedative treatments (13% at inclusion versus 4% at M6) was observed in partners. Finally, 39% complained of leaks due to poor interface adjustment, but only 2% complained of noise from the CPAP device. During follow‐up, the 4 dyads who had separate rooms for 1 to 3 nights a week were sleeping in the same bed again.

**TABLE 4:** Sleep quality of partners and OSA patients at baseline (M0), after 2 (M2) and 6 months (M6) of CPAP treatment.

	Partners			Patients		
	M0	M2	M6	M0	M2	M6
AIS	6 [3–8.8]	4 [2–7] *	4 [3–6] *	9.5 [6–13]	3.5 [2.8–8] **	3 [2–6.8] **
ESS	6 [3–9]	5 [2.3–7] *	4 [2–7.8]	11.5 [9–15]	8 [5–11] **	7 [3–8] **
LSEQ		25 [13–35.5]	23 [12.3–31]		23 [13.3–35]	25 [10.5–35.5]
PSQI	5.5 [3.3–7]	4.5 [3–7]	5 [3–7.8]	6 [4–9]	5 [3–7.8] *	4.5 [3–6] *

Abbreviations: AIS, Athens insomnia scale; ESS, Epworth sleepiness scale; LSEQ, Leeds sleep evaluation questionnaire; M0, at baseline; M2, after 2 months of treatment; M6, after 6 months of treatment; PSQI, Pittsburgh sleep quality index.

* *p* < 0.05.

** *p* < 0.001, quantitative values expressed as medians [25th—75th percentile].

**TABLE 5:** Comparison of VAS regarding sleep completed by partners and OSA patients at baseline (M0), after 2 (M2) and 6 months (M6) of CPAP treatment.

	M0	M2	M6
VAS (0 to 10) completed by patients			
Do you think you are affecting your partner's quality of sleep?	3 [2–4]	1 [0–3.8] **	1 [0–2] *
Do you think your partner is concerned about your OSA?	3 [2–7]	1 [0–4] **	1 [0–5] **
Do you feel the benefits of your CPAP treatment?		8 [6.3–9.8]	7 [5–10]
Are you satisfied with your CPAP treatment?		8 [6.3–10]	8 [5–10]
Do you need your partner's help in setting up your CPAP treatment?		0 [0–0]	0 [0–0]
VAS (0 to 10) completed by partners			
How much does your partner disturb your sleep?	2 [1–4]	0 [0–2] **	1 [0–2.8] *
Do you feel concerned about your partner's OSA?	3 [2–5]	0 [0–2] **	0 [0–1] **
Do you feel concerned about your partner's CPAP?		0 [0–2]	0 [0–1.5]
Do you benefit from your partner's CPAP?		7 [5–9.8]	5 [1.3–8]
Do you sleep better since your partner's CPAP?		8.5 [5–10]	6 [3–9]
Are you satisfied with your partner's CPAP treatment?		8 [7–10]	8 [5.3–10]
Does your partner need your help in setting up his CPAP treatment?		2 [0–3]	10 [10–10]

Abbreviations: CPAP, continuous positive airway pressure; M0, at baseline; M2, after 2 months of treatment; M6, after 6 months of treatment; OSA, obstructive sleep apnea; VAS, visual analgesic scale.

* *p* < 0.05.

** *p* < 0.001, quantitative values expressed as medians [25th—75th percentile].

### 3.5 Quality of Life

Table 6 presents the results of the quality‐of‐life assessment during follow‐up. There was a significant improvement in the ‘energy/vitality’, ‘mental health’ and ‘social functioning’ domains of the SF‐36 questionnaire for both apneic patients and partners.

**TABLE 6:** Quality of life and psychological state of partners and OSA patients at baseline (M0), after 2 (M2) and 6 months (M6) of CPAP treatment.

	Partners			Patients		
	M0	M2	M6	M0	M2	M6
SF36‐ Physical functioning	88 [70–95]	90 [71–95]	90 [70–99]	78 [56–90]	75 [56–89]	80 [56–90]
SF36‐ Physical limitations	100 [50–100]	100 [50–100]	100 [75–100]	50 [0–75]	75 [31–100] **	100 [50–100] *
SF36‐ Emotional limitations	100 [33–100]	100 [42–100]	100 [67–100]	67 [8–100]	100 [42–100] *	100 [67–100]
SF36‐ Energy/vitality	55 [45–69]	65 [55–80] **	60 [50–75] *	40 [26–54]	55 [40–65] **	55 [40–70] *
SF36‐ Mental health	68 [49–76]	76 [60–87] **	74 [61–86] *	58 [45–76]	68 [52–80] *	72 [53–87] *
SF36‐ Social functioning	75 [63–89]	88 [63–100] **	88 [63–100]	63 [50–75]	75 [63–100] **	88 [63–100] *
SF36‐ Pain	68 [45–89]	78 [58–90] *	78 [57–90]	50 [35–68]	68 [48–90] **	68 [48–98] **
SF36‐ General health perception	55 [45–70]	65 [50–79] *	65 [41–79] *	53 [45–70]	55 [45–74]	56 [50–75]
HADS‐anxiety	7 [5–11]	6 [3–9] **	4.5 [2.3–9] **	7 [5–10]	5 [3–8] *	4.5 [3–7] *
HADS‐depression	3.5 [2–6]	2 [1–5] *	2 [1–5.8] *	5 [2.3–7]	4 [2–7] *	3 [1–6.8]

Abbreviations: HADS, hospital anxiety and depression scale; SF‐36, short form 36 health survey.

* *p* < 0.05.

** *p* < 0.001, quantitative values expressed as medians [25th—75th percentile].

Concomitantly, the scores of the ‘anxiety’ and ‘depression’ sub‐domain of the HADS questionnaire significantly improved for both patients and partners.

In univariate analysis, there was no significant correlation between improvement in DAS‐16 and age, length of the relationship, mattress width, OSA severity at diagnosis, CPAP efficacy (compliance, residual AHI, unintentional leaks evaluated at M2) or improvement in self‐assessed sleep quality (AIS) and sleepiness (ESS) in both groups.

### 3.6 Principal Component Analysis

**FIGURE 3:**
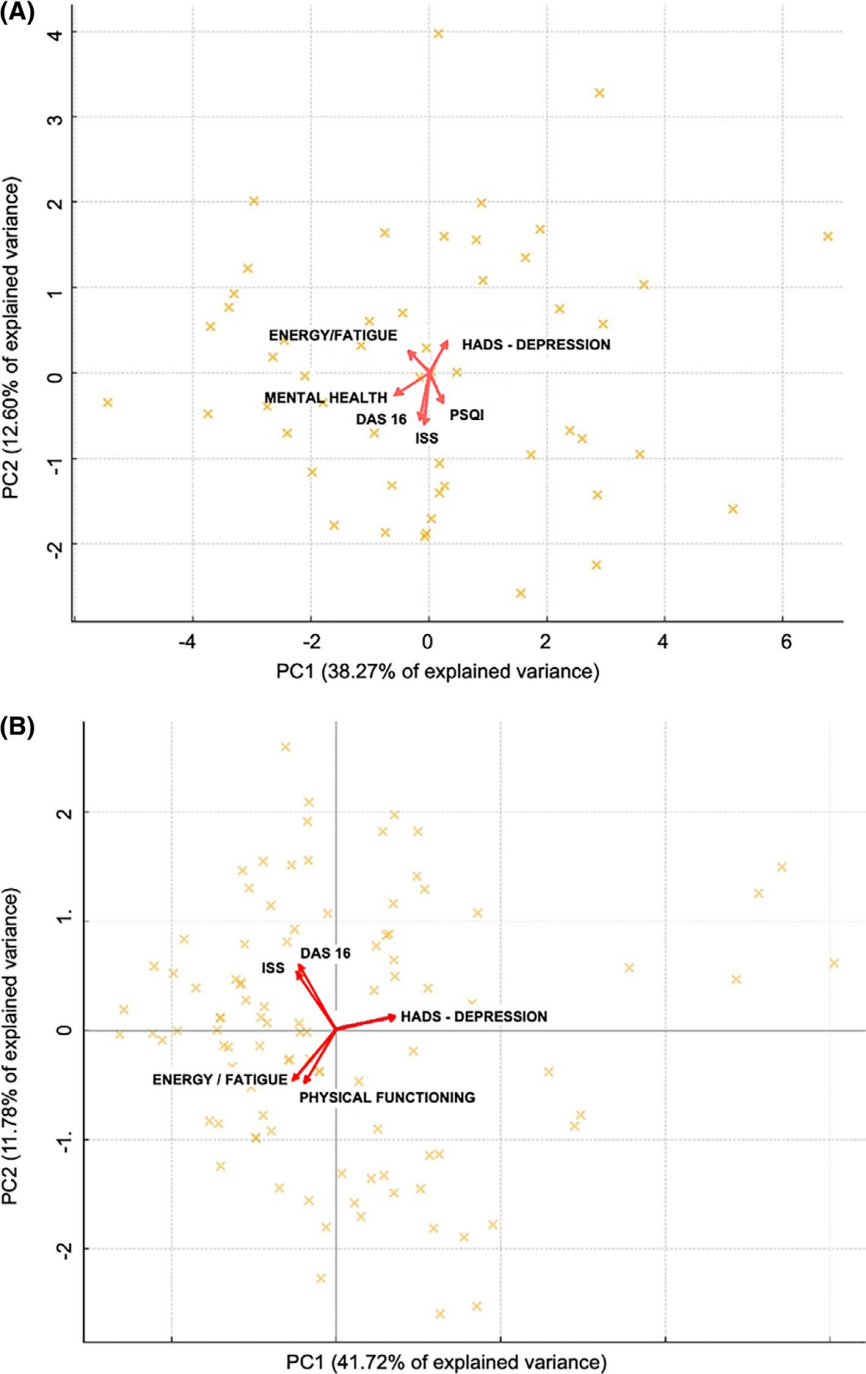
Biplot of variables contributing the most to the first two principal components PC1 and PC2 in patients (A) and in partners (B).

Tables 7 and 8 summarise the PCA in patients and in partners, respectively. PC1 and PC2 explain about 50% of the variance. PC1 is influenced almost equally by all variables, reflecting the complex and multifactorial change in health status under CPAP. PC2 is primarily driven by DAS‐16 and ISS scores. PC1 and PC2 are represented in biplots for patients (Figure 3A) and partners (Figure 3B). In both groups, DAS‐16 and ISS are among the top five variables with the highest vector weights, moving together in the same direction. This suggests a link between the dyadic relationship and sexual satisfaction.

**TABLE 7:** Participation of variables in the different principal components in patients.

	PC1	PC2	PC3	PC4	PC5
Explained variance (%)	38.27	12.60	9.86	6.79	6.63
Cumulative variance (%)	38.27	50.87	60.73	67.52	74.14
Contribution of variables for each principal component					
HADS‐ depression	0.29	0.35	−0.12	−0.01	0.13
HADS‐ anxiety	0.26	0.04	0.41	0.02	0.28
ESS	0.16	−0.11	−0.09	0.780	0.22
ISS	−0.13	−0.51	−0.24	0.05	0.37
AIS	0.32	−0.15	0.29	−0.13	0.10
PSQI	0.19	−0.29	0.49	−0.14	−0.00
DAS‐16	−0.08	−0.61	0.03	−0.02	0.01
SF36‐ Social functioning	−0.27	0.08	0.30	−0.07	0.46
SF36‐ Physical limitations	−0.26	0.10	0.43	0.40	−0.14
SF36‐ Emotional limitations	−0.30	−0.10	0.23	0.12	−0.30
SF36‐ Energy/vitality	−0.30	0.21	0.06	0.21	0.08
SF36‐ Mental health	−0.33	−0.16	−0.17	−0.03	−0.20
SF36‐ Social functioning	−0.34	0.07	0.18	−0.26	−0.04
SF36‐ Pain	−0.26	0.14	0.10	0.04	0.25
SF36‐ General health perception	−0,23	0.08	−0.14	−0.18	0.53

Abbreviations: AIS, Athens insomnia scale; DAS‐16, dyadic adjustment scale in 16 questions; ESS, Epworth sleepiness scale; HADS, hospital anxiety and depression scale; ISS, index of sexual satisfaction; PSQI, Pittsburgh sleep quality index; SF‐36, short form 36 health survey.

**TABLE 8:** Participation of variables in the different principal components in partners.

	PC1	PC2	PC3	PC4	PC5
Explained variance (%)	41.72	11.78	7.50	6.57	6.53
Cumulative variance (%)	41.72	53.50	61.00	67.57	74.09
Contribution of variables for each principal component					
ESS	0.13	0.29	−0.64	0.17	0.32
ISS	−0.18	0.54	0.80	−0.28	0.12
HADS‐ depression	0.31	−0.16	−0.27	−0.13	0.03
DAS‐16	−0.15	0.60	0.12	−0.09	0.18
AIS	0.25	0.30	−0.08	0.00	−0.52
PSQI	0.23	0.23	−0.05	−0.05	−0.61
SF36‐ Energy/vitality	−0.31	0.09	−0.20	0.48	−0.19
SF36‐ Emotional limitations	−0.33	−0.06	0.16	−0.01	−0.01
SF36‐ Physical limitations	−0.22	−0.11	−0.16	−0.76	−0.11
SF36‐ Physical functioning	−0.29	−0.09	−0.41	−0.14	−0.22
SF36‐ Mental health	−0.34	0.11	−0.22	0.10	0.05
SF36‐ Social functioning	−0.30	−0.08	0.13	0.01	0.00
SF36‐ Pain	−0.30	0.05	0.23	0.19	−0.30
SF36‐ General health perception	−0,30	−0.19	−0.33	0.01	−0.15

Abbreviations: AIS, Athens insomnia scale; DAS‐16, dyadic adjustment scale in 16 questions; ESS, Epworth sleepiness scale; HADS, hospital anxiety and depression scale; ISS, index of sexual satisfaction; PSQI, Pittsburgh sleep quality index; SF‐36, short form 36 health survey.

Biplots illustrate the importance of two other aspects: (i) sleep quality, as evaluated by the SF36‐energy/vitality, SF36‐physical functioning and PSQI, and (ii) the emotional component, as evaluated by the HADS‐depression and SF36‐mental health, with differences in factor loading between patients and their partners. (i) Sleep quality evolves in the same way as SF36‐energy/vitality, SF36‐physical functioning, and the PSQI score. (ii) The emotional component evolves similarly to HADS‐depression and SF36‐mental health. Taken together, these results suggest that the dyadic relationship and sexual satisfaction increase with psychological well‐being and sleep quality. We found that the progression of patients treated for sleep apnea is influenced by emotional factors, as evidenced by the strong involvement of the ‘HADS depression’ and ‘SF‐36 mental health’ variables in the first two principal components (PC1 and PC2). In contrast, the progression of the partners appears to be more influenced by physical factors, with the ‘SF‐36 physical functioning’ variables and ‘SF‐36 energy/fatigue’ playing a more significant role in PC1 and PC2.

## 4 Discussion

To the best of our knowledge, this is the first study to demonstrate objective improvements in the dyadic relationship (assessed by the DAS‐16) and sexual satisfaction (assessed by the ISS) in both OSA patients and partners after 2 months of CPAP treatment, with a persistent effect after 6 months of treatment. Analysis of sleep quality assessed by several scores also showed significant improvement in both partners, as most domains of the SF‐36 quality of life questionnaire and HADS scores. VAS score suggested that partners were less bothered by apneic sleep disturbance, worried less about nocturnal respiratory arrests, and experienced little discomfort related to the CPAP device.

### 4.1 Effects of CPAP on the Dyadic Relationship

Poor sleep quality may contribute to marital dissatisfaction and increase conflict within the couple (Luyster 2017; Troxel et al. 2007; Ye et al. 2017). Only a few studies have looked at marital relationships after initiation of CPAP treatment. Their results were divergent, probably due to the small number of subjects studied, different assessment methods or high marital satisfaction at baseline (Luyster 2017).

The DAS‐16 is mainly used to assess the quality of couple relationships in the field of human sciences, investigating both the degree of agreement and the quality of interactions within the couple (Antoine et al. 2008). Overall, the couples included in our study had a good marital relationship with high DAS‐16 scores at baseline, well above the ‘no difficulty’ threshold of 54 points (Antoine et al. 2008). The improvement of +1 [−1; 3] and +2 [−1; 5] points in the DAS‐16 observed in both OSA patients and partners after 2 months of CPAP treatment is consistent with the results of a positive psychological intervention in a cohort of couples who already had a good level of functioning (Antoine et al. 2020). The symmetrical improvement in the DAS‐16 scores in both OSA patients and partners suggests that changes in the behaviour of one partner may induce changes in the behaviour of the other (Hilpert et al. 2016).

Moreover, the concomitant improvement in ISS is consistent with the DAS‐16 results. There is a close interaction between the quality of the couple's relationship and sexual satisfaction (Trudel 2011). A positive dyadic relationship is predictive of high sexual satisfaction (Brown and Weigel 2018), and sexual satisfaction increases with high dyadic adjustment, low marital conflict, and frequent sharing of common activities (Zaia, n.d.). Sexual dysfunction is common in patients with OSA (Liu et al. 2015; Zaia, n.d.). In men, CPAP treatment may improve erectile dysfunction by reducing intermittent hypoxia, hyperadrenergic response and chronic endothelial damage (Stilo et al. 2023; Mittleman et al. 2014). Improved daytime functioning in both partners and patients, through a significant reduction in sleepiness, anxiety and depression (Laharnar et al. 2024), may also contribute to sexual fulfilment (Lai et al. 2016). Furthermore, Laharnar et al. (2024) suggest that nocturnal CPAP does not affect intimacy. Here, we found no correlation between DAS‐16 score and the CPAP device.

### 4.2 Effects of CPAP on Sleep and Daytime Functioning

Several studies have found that partners of OSA patients frequently seek medical advice for sleep disorders (Rosa et al. 2022; Olsen et al. 2010). Nocturnal OSA symptoms have an impact on partners (Luyster 2017; Strawbridge et al. 2004), who report monitoring the patient for respiratory arrests or being bothered by snoring, sleep‐related choking, tossing or frequent waking for urination (Luyster 2017). Insomnia is three times more frequent and daytime sleepiness is two times more frequent in partners of OSA patients than in the general population, even after adjusting for age, BMI, number of children under 18, working hours and use of sleeping pills (Ulfberg et al. 2000).

As expected, CPAP use improves sleep in OSA patients, but the improvement depends on consistent use of the device. In our study, adherence to treatment was relatively better than data available in the literature (Benjafield et al. 2019), likely thanks to the personalised therapeutic education session dispensed by a trained nurse on an outpatient basis. During follow‐up, partners' sleep quality also improved. The median AIS score for partners dropped from 6 to 4 points (*p* = 0.007). This suggests a clinically relevant difference at M2, since the threshold for insomnia is 6 points (Soldatos et al. 2000). The median LSEQ score increased by 25 points, indicating an improvement under treatment (Zisapel and Laudon 2003). Daytime sleepiness also improved both in patients and in partners, even if they were less symptomatic at baseline. Untreated OSA was associated with poor physical and mental health (Ye et al. 2017; Hilpert et al. 2016; Trudel 2011). Our results show an improvement in quality of life in OSA patients, in line with existing literature (Batool‐Anwar et al. 2016; Siccoli et al. 2008), but also in their bedpartners, which is more controversial (Luyster 2017; Labarca et al. 2020; Poletti et al. 2025). Regarding mental health, HAD scores decreased significantly in both patients and partners. Previous research has shown that the prevalence of anxiety and depression is higher in OSA patients, especially those who are single or have sexual dysfunction (Gharsalli et al. 2022).

### 4.3 Importance of Partners in the Experience and Management of Chronic Disease

There is a growing body of evidence in the literature on the influence of partners on patients' health behaviours (Gharsalli et al. 2022). Partner involvement can have a profound effect on whether patients try CPAP and use it regularly (Baron et al. 2009, 2011; Ye et al. 2017; Broström et al. 2010; Hoy et al. 1999; Luyster et al. 2016; Elfström et al. 2012). Collaborative involvement of partners is an important factor in adherence to CPAP, particularly in the first 6 months (Batool‐Anwar et al. 2017; Budhiraja et al. 2010). Similarly, a stable relationship of over 30 years, with a strong relationship, is also a factor positively influencing adherence to CPAP (Gentina et al. 2019).

Beyond compliance, several studies have looked at the benefits of couple‐oriented interventions in the context of chronic diseases, demonstrating significant improvements in psychological and marital functioning (Luyster 2017; Ye et al. 2017). Therefore, it seems essential to fully integrate willing partners in OSA management, from diagnosis onwards (Ye et al. 2017; Laharnar et al. 2024).

### 4.4 Study Limitations

Our study has several limitations, mainly its monocentric nature and possible recruitment bias. The participating dyads are (i) motivated to use CPAP, (ii) available for joint appointments, with a predominance of retirees and people who live near the Dijon University Hospital, and (iii) comfortable with issues related to their sexuality. Even in a medical context, sex is considered a taboo subject, and some patients or partners do not wish to talk about it out of modesty or shame (Zaia, n.d.). Others, on the contrary, reported that they found sexuality and intimacy to be important subjects during treatment. Finally, we only included OSA patients eligible for CPAP treatment according to current French guidelines (Revue des Maladies Respiratoires 2010).

### 4.5 Perspectives

This study provides a better understanding of the management of OSA from a dyadic perspective. Emphasis should be placed on the engagement of partners throughout the diagnosis and treatment of OSA, and the importance of educating them about the negative health effects of OSA and the benefits of CPAP treatment. It appears that a collaborative approach may be beneficial for both patients and partners. In future, a qualitative study may be an opportunity to better understand the impact of OSA on partners and partners' role in CPAP adhesion. Exploring the individual perspectives of the OSA patient and their partner, especially the beliefs and concerns regarding the CPAP device prior to use, could be a basis for a new education programme for CPAP users, including a couple‐based intervention to promote CPAP adherence.

## Author Contributions

**J. Chorvoz:** conceptualization, investigation, writing – original draft, methodology, validation, visualization, writing – review and editing, software, formal analysis, project administration, data curation, supervision, resources. **C. Rabec:** conceptualization, investigation, methodology, validation, visualization, writing – review and editing, supervision. **M. Labruyère:** methodology, formal analysis, writing – review and editing, software, data curation. **H. Devilliers:** methodology, writing – review and editing, formal analysis, software. **P. Sabot:** investigation, project administration, writing – review and editing. **A. Berrier:** investigation, writing – review and editing. **D. Schenesse:** writing – review and editing. **P. Mouillot:** project administration, writing – review and editing. **P. Bonniaud:** writing – review and editing, conceptualization, methodology, validation, supervision. **M. Georges:** investigation, conceptualization, methodology, validation, visualization, writing – review and editing, software, formal analysis, project administration, supervision, resources.

## Conflicts of Interest

The authors declare no conflicts of interest.

## Data Availability

The data that support the findings of this study are available on request from the corresponding author. The data are not publicly available due to privacy or ethical restrictions.
